# Psychiatry

**Published:** 1911-05

**Authors:** Ralph Reed


### Psychiatry.

### PHILISTINE AND GENIUS.

It is now 9 a. m. on a weekday. All over the United States thousands of children are busy with their morning lessons. We almost said lesson, for in fact a large proportion of those thousands are busy with the same lesson. Today we are constantly preached the gospel of efficiency. Efficiency means systematization, precision, habit—in the schools, in brief, the reduction of a very beautiful thing—the education of a little child to the basis of our modern machine system. And that is what education is today—very largely a machine process. Routine is king; uniformity the supreme desideratum.

Dr. Boris Sidis, in a recent address delivered before the summer school commencement exercises at Harvard (*Monthly Encyclopedia and Medical Bulletin*), with the title "Philistine and Genius," laments the present state of affairs in education, and attempts to inspire the teachers he addresses with a better view of the real meaning of education.

"In every work the beginning is the most important part, and the groundwork of a man's character is laid in his childhood," remarks Sidis. He does not approve of the conventional nursery education of the child. "Not only do we neglect to lay the necessary solid basis in the early life of the child," but, he says—

"We do not even take care to clear the ground. In fact, we even make the child's soul a dunghill, full of vermin, of superstition, possessed by hosts of ghosts of fears and prejudices—a hideous heap saturated with the spirit of credulity. We regard the child's mind as a *tabula rasa,* a vacant lot, and empty on it all our rubbish and refuse. We labor under the delusion that stories and fairy tales, myths and deceptions about life and man, are good for the child's mind. Is it a wonder that on such a foundation men can only put up shacks and shanties? We forget the simple fact that what is harmful for the adult is still more harmful to the chiid. Surely what is poisonous to the grown-up mind can not be useful food to the young. If credulity in old wives' tales, lack of individuality, sheepish submissiveness, barrack discipline, unquestioned, uncritical belief in authority, meaningless imitation of jingles and gibberish, memorization of Mother Goose wisdom, repetition of incomprehensible prayers and articles of creed, unintelligent aping of good manners, silly games, prejudices and superstitions and fears of the supernormal and supernatural, are censured in adults, why should we approve their cultivation in the young?"

The author now digresses in order to take exception to the frothy philosophies of optimism which are today so prevalent:

"We are confirmed optimists, and sow optimism broadcast. We have optimistic clubs, mental scientists and Christian scientists—all afflicted with incurable ophthalmia to surrounding evil and misery. We are scientific, we are evolutionists, we have faith in the sort of optimism taught by Leibnitz in his famous Theodicea. We are the Candids of our oracles, the Panglosses. You may possibly remember what Voltaire writes of Professor Pangloss: 'Pangloss used to teach the science of metaphysico-theologo-cosmologo-noodleology. He demonstrated to admiration that there is no effect without a cause, and that this is the best of all possible worlds. It has been proved, said Pangloss, that things can not be otherwise than they are; for everything the end for which everything is made is necessarily the best end. Observe how noses are made to carry spectacles, and spectacles we have accordingly. Everything that is, is the best that could possibly be.' It is such shallow optimism that now gains currency. Verily, we are stricken with mental blindness.

"The favored type of superintendent of our public education is such a hopeless philistine, but possessed of all the conceit of the mediocre business man. Routine is his ideal. * * * Their merit is routine, discipline and the hiring of cheap employes. It is certainly unfortunate that a good number of our would-be scientific pedagogues are such hopeless philistines, with so absurd an exaggeration of themselves that they are well satisfied if the mass of their pupils turn out as exact reproductions of themselves. What can be expected of a nation that entrusts the fate of its young generation to the care and carelessness of young girls, to the ire of old maids, and to the pettifogging officials with their educational red tape discipline and routine?"

He gives the teachers this good advice:

"You have heard the psychologizing educator advise the formation of good, fixed, stable habits in early life. Now I want to warn you of the dangers of such unrestricted advice. Fixed adaptations, stable habits, tend to raise the thresholds of mental life, tend to inhibit the liberation, the output of reserve energy. Avoid routine. Do not let your pupils fall into the ruts of habits and customs. Do not let the best of habits harden beyond the point of possible modification. * * * The important thing in education is not so much the formation of habits as the power of their reformation. * * * The cultivation of the power of habit-disintegration is what constitutes the proper education of man's genius."

We have been able to quote all too little of this splendid address. We wish that a copy of it could be placed in the hands of every teacher and school superintendent in the United States, although for the latter much of it would not be pleasant reading.—Ralph Reed, M. D., in *The Lancet-Clinic*.

